# I Like the Way You Eat It: Lemur (*Indri indri*) Gut Mycobiome and Geophagy

**DOI:** 10.1007/s00248-020-01677-5

**Published:** 2021-01-20

**Authors:** Luigimaria Borruso, Alice Checcucci, Valeria Torti, Federico Correa, Camillo Sandri, Daine Luise, Luciano Cavani, Monica Modesto, Caterina Spiezio, Tanja Mimmo, Stefano Cesco, Maura Di Vito, Francesca Bugli, Rose M. Randrianarison, Marco Gamba, Nianja J. Rarojoson, Cesare Avesani Zaborra, Paola Mattarelli, Paolo Trevisi, Cristina Giacoma

**Affiliations:** 1 Faculty of Science and Technology, Free University of Bolzano-Bozen, Piazza Università 5, 39100 Bolzano-Bozen, Italy; 2 Department of Agricultural and Food Sciences, University of Bologna, Viale Fanin 44, 40127 Bologna, Italy; 3 Department of Life Sciences and Systems Biology, University of Torino, Torino, Italy; 4 Department of Animal Health Care and Management, Parco Natura Viva - Garda Zoological Park, Bussolengo, Verona, Italy; 5 Dipartimento di Scienze Biotecnologiche di Base, Cliniche Intensivologiche e Perioperatorie, Università Cattolica del Sacro Cuore, Largo A. Gemelli 8, 00168 Rome, Italy; 6 Dipartimento di Scienze di Laboratorio e Infettivologiche, Fondazione Policlinico Universitario A. Gemelli IRCCS, Largo A. Gemelli 8, 00168 Rome, Italy; 7 Groupe d’Étude et de Recherche sur les Primates de Madagascar (GERP), Cité des Professeurs, Fort Duchesne, BP 779, 101 Antananarivo, Madagascar; 8 Mention d’Anthropobiologie et de Développement Durable (MADD), Université de Antananarivo, Antananarivo, Madagascar; 9 Laboratoire de Pédologie, FOFIFA à Tsimbazaza, BP.1690 Antananarivo, Madagascar

**Keywords:** Mycobiome, Gut, Soil quality, Non-human primates, Conservation, *Indri indri*

## Abstract

Here, we investigated the possible linkages among geophagy, soil characteristics, and gut mycobiome of indri (*Indri indri*), an endangered lemur species able to survive only in wild conditions. The soil eaten by indri resulted in enriched secondary oxide-hydroxides and clays, together with a high concentration of specific essential micronutrients. This could partially explain the role of the soil in detoxification and as a nutrient supply. Besides, we found that soil subject to geophagy and indris’ faeces shared about 8.9% of the fungal OTUs. Also, several genera (e.g. *Fusarium*, *Aspergillus* and *Penicillium*) commonly associated with soil and plant material were found in both geophagic soil and indri samples. On the contrary, some taxa with pathogenic potentials, such as *Cryptococcus,* were only found in indri samples. Further, many saprotrophs and plant-associated fungal taxa were detected in the indri faeces. These fungal species may be involved in the digestion processes of leaves and could have a beneficial role in their health. In conclusion, we found an intimate connection between gut mycobiome and soil, highlighting, once again, the potential consequent impacts on the wider habitat.

**Supplementary Information:**

The online version contains supplementary material available at 10.1007/s00248-020-01677-5.

## Introduction

Geophagy, the intentional consumption of soil, is practiced by many different human cultures over different continents [1]. Cultural tradition, together with sensory trap, hunger or stress relief, are the main three non-adaptive explanations for human geophagy [1]. The well-documented occurrence of geophagic behaviour in many vertebrates, including non-human Primates, encourages the formulation of two main adaptive hypotheses: (i) the supplementation function consisting of supplementing microelements that are lacking in the diet and (ii) the protective function of soil in pH regulation, against toxins and parasites [2]. In this respect, lemurs’ radiation in more than 100 species, colonizing different habitats and performing soil eating in at least 40 species, make them a very promising model for untangling the causes and functional consequences of geophagy [3]. Indri (*Indri indri*), the biggest among living lemurs, has a well-documented feeding and geophagy behaviour. This lemurs species is classified as ‘critically endangered’ by the IUCN Red List of Threatened Species due to the destruction and fragmentation of its habitat [4]. Furthermore, indris have never been successfully bred in captivity [5]. This evidence suggests that some behavioural and environmental factors are not satisfied with the already tested captivity protocols.

**Fig. 1:**
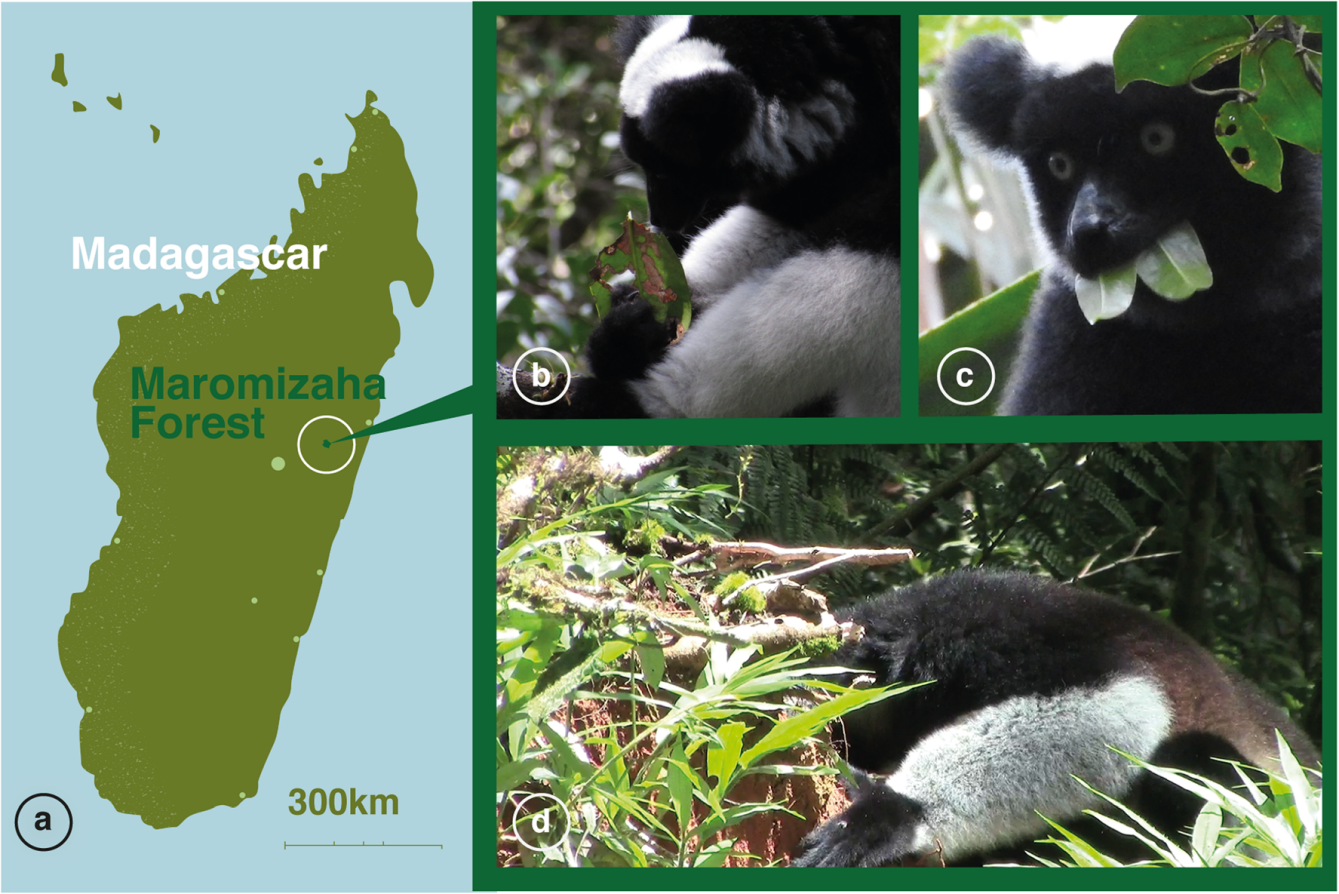
a Localisation of the study site, the Maromizaha Forest, in north-eastern Madagascar; (**b**) indri eating a mature, speckled leaf; (**c**) indri eating young leaves; (**d**) indri eating soil from a fallen tree site

The indris’ diet is mainly folivorous (i.e. immature leaves), but it may include even bark, seeds, flowers and fruits [5] (Fig. 1 b, c and d, Supplementary Video S1). In the folivorous diet, the interactions between host and gut microbiome are necessary for the processes involved in cellulase activities due to the absence of these enzymes in all vertebrates [6, 7]. Primates’ gut harbour a plethora of microbes, including archaea, bacteria and fungi, which play a crucial role in the digestion process, health and behaviour [8–11]. Despite the limited studies on primate mycobiome, indications are now accumulated on the fungi’s important role in host physiology [12, 13]. However, there is evidence that the primates’ gut lacks a stable core mycobiome, unlike the bacterial microbiome [14]. In this respect, to define if a fungal species inhabits the gut stably or transiently remains an open question. The high inter-individual variability can be explained considering that diet, geography and environment are the primary drivers in shaping the mycobiome composition [10] and fungal species of environmental or food-associated origin could transiently colonise the gut influencing the mycobiome composition. In this regard, we aimed to investigate the linkages between geophagic soil and indris gut mycobiome, using samples collected in the Maromizaha forest (Madagascar) (Fig. 1a). Specifically, we evaluated (i) the possible role of the soil properties eaten by indris and (ii) the putative geophagic soil contribution to the fungal communities inhabiting the indris’ gut.

ESM 3(MP4 123036 kb)

## Material and Methods

### Behavioural Observation, Faecal and Soil Sample Collection

All the samples were obtained from indris (faecal material) and geophagic soils in Maromizaha forest (latitude 18° 57′ S and 19° 00′ S, longitude 48° 26′ E and 48° 31′ E, Madagascar) between December 4 and 6, 2018 (Fig. 1a). Indris has been the subject of ongoing etho-ecological studies since 2009 [15]. Records are kept as photographs or videos.

Faecal samples were collected from 9 individuals (Table 1) following the groups’ activity patterns in their natural habitat. Every single individual was recognised by natural trough marks [16]. We collected faecal samples immediately after defecation, when only one animal was present, to avoid individual misidentification during the sampling process [15]. Disposable sterile gloves were worn when collecting samples to prevent contamination. Specifically, about 5 g of faeces was collected into screw-capped tubes, with an integrated plastic shovel-like tool attached to the cap, containing 10 ml of RNAlater (Thermofisher, Waltham, MA, USA). The stool amount was taken from the middle of each fresh piece of faeces to avoid soil contamination. Next, the small plastic shovel-like tool attached to the cap of screw-capped tubes was used to scoop faecal samples. Every container was sealed immediately after the collection to avoid cross-contamination among samples. Seven soil samples (Table 1) were collected from the seven geophagy sites. All samples have been preserved in a portable refrigerator and then stored at − 20 °C in the laboratory until downstream analysis.

**Table 1:** Information of the indris and soils considered

Name	Group	Age (years)	Sex	Geophagic behaviour
Bemasoandro	8MZ	> 6	Female	Yes
Emè	8MZ	1	Male	Yes
Zafy	8MZ	6	Male	Yes
Eva	4MZ	> 6	Female	Yes
Koto	4MZ	> 6	Male	Yes
Mahagaga	3MZ	> 6	Male	Yes
Bevolo	1MZ	> 6	Female	Yes
Cami	1MZ	1	Female	Yes
Dary	2MZ	6 months	Unknown	Yes

### Soil Characterisation

Soil samples were air-dried, milled and sieved at 2 mm for soil analysis in agreement with SSSA methods [17]. Briefly, total carbon (C_tot_) and total nitrogen (N_tot_) were determined using an elemental analyser (Flash 2000, Thermo Scientific, Germany) coupled with an isotopic mass spectrometer (DELTA Advantage, Thermo Scientific, Germany). Pseudo total element concentration was determined after acid mineralisation with aqua regia and hydrogen peroxide in an Ethos TC microwave lab station (Milestone, Bergamo, Italy) by an inductively coupled plasma optical emission spectrometer (ICP-OES, Ametek Spectro, Arcos, Germany). Iron, aluminium, titanium and silica oxide concentrations were determined by ICP-OES (Ametek Spectro, Arcos, Germany) after extraction with sodium dithionite (Na_2_S_2_O_4_).

### DNA Extraction and NGS Sequencing

Total DNA extraction from 200 mg faecal and soil samples was carried out using the DNeasy PowerSoil Kit (QIAGEN, Hilden, Germany) with a modification to the protocol including a pre-treatment with lyticase. Briefly, the samples were initially treated with 200 U lyticase (Sigma-Aldrich Co., Gillingham, UK), homogenised and incubated for 30 min at room temperature [18]. Lastly, the DNA was eluted twice to improve yield. Extracted DNA was quantified using a QuBit 2.0 Fluorometer Assay (Life Technologies Corporation) and then adjusted at 1 ng μL^−1^.

Fungal ITS region was amplified using the primer pairs ITS3 (5′-TCGTCGGCAGCGTCAGATGTGTATAAGAGACAGGCATCGATGAAGAACGCAGC-3′) and ITS4 (5′-GTCTCGTGGGCTCGGAGATGTGTATAAGAGACAGTCCTCCGCTTATTGATATGC 3′) modified with the required Illumina sequencing adaptors [19]. PCR was conducted in a total reaction volume of 25 μl using the Platinum™ Taq DNA Polymerase High Fidelity (Thermo Fisher Scientific, Italy), 1 μl of each primer (10 μM) and 2.5 μL of DNA template. In all samples, 0.4 mg/ml BSA was added. The thermal cycling protocol consisted of 94 °C for 2 min followed by 30 cycles each of 30 s at 94 °C, 30 s at 53 °C and 30 s at 72 °C and final elongation at 72 °C for 5 min. The libraries were prepared by BMR-Genomics Ltd. (http://www.bmr-genomics.it/) and sequenced on the MiSeq platform (Illumina Inc., San Diego, Ca, USA).

### Bioinformatics Analysis and Statistical Analysis

Raw data were quality checked via FastQC [20]. Sequences were pre-processed, quality filtered, trimmed, de-noised, merged, modelled and analysed via DADA2 within QIIME2 [21]. Chimeras were discarded using the ‘consensus’ method [22]. Finally, the sequences variants were clustered using VSEARCH with a cut-off of 97% [23]. The taxonomy annotation was performed using a Naïve-Bayes classifier trained on the UNITE+INSD database against the representative sequences [24]. The taxonomic annotated OTU table was parsed against the FunGuild (v1.0) database to assign putative functional guilds to each sample [25]. All sequences have been submitted to the European Nucleotide Archive (EMBL-EBI) under the study accession number PRJEB39443 (sample accession number from ERS4827963 to ERS4827978). *Cryptococcus* sequences were aligned using CLUSTALW [26]. For phylogenetic reconstruction, the neighbour-joining algorithm and Kimura’s two-parameter model were used with complete deletion of positions containing gaps or missing data and 1000 bootstrap replications [27]. Phylogenetic analyses were carried out in MEGAX version 10.2 [27].

Rarefaction curves and Venn diagram were created using ‘ggplot’ and ‘vegan’ packages within the ‘R’ environment [28–30]. Linear discriminant analysis effect size (LEfSe) algorithm (considering an LDA score ≥ 2 and *p* value < 0.05) was applied to discover the most abundant fungal genera (average > 0.3%) and functional guilds associated with indri and soil samples [31]. All the analyses were performed on rarefied data to 1154 reads.

## Results and Discussion

### Geophagy

In all nine individuals considered in this study (Table 1), we observed soil eating behaviour and a quite stereotypical ingestion method (Fig. 1d; Supplemental Video S1). The focal group always moved to a precise location solely for soil consumption. In all soil feeding-bouts observed, an indri descended first to the ground, jumping from a tree or a liana near the geophagy site, and started to eat soil. During one soil feeding-bout, one member of the group began to eat. The other members approached the site and stayed on the nearest trees monitoring the surrounding environment (< 10 min) till he/she left the site, and a new indri took his/her turn in eating soil, one after the other.

The individuals consumed the soil directly by eating the exposed horizons with the mouth or collecting a small amount of soil with the hand and successively introducing it into the mouth.

When the mother carried the babies (i.e. Eme and Cami) (Table 1) and the female entered the site, we observed geophagy also in the youngest animals (Supplemental Video S1). After all individuals had fed, the group scurried out of the geophagy site. Next, the group reached a new location for eating or resting. Geophagy sites observed were mostly in the proximity of fallen trees, landslides or soft mounds of earth, revealing the lower soil horizons. There were exposed soils at the bases of trees uprooted by wind or rainfall in the valley, at lower elevations, in the slopes. All the locations were relatively free of debris (grass, leaves, stones, etc.) (Supplementary Video S1).

### Geophagic Soil Composition

Soil composition analysis revealed that the different sampling sites might be classified as Oxisols rich in secondary oxide-hydroxides and highly weathered clays [32]. In particular, soil analysis revealed that the sandy loam was characterised by a quite acid pH, relatively rich in organic carbon, total nitrogen, potassium and magnesium, but poor in phosphorous and calcium (Table 2). Soil components as secondary oxide-hydroxides are characterised by a high specific surface area, being thus ideal candidates for the gut detoxification of indri. This type of soil could be involved in the plant toxin adsorption, such as tannins, terpenes and cyanogenic glycosides derived from the diet based on immature fruits and leaves [2, 33]. Further, the low pH is a common characteristic of geophagic soils [34]; a consequent higher metal availability could be advantageous for their incorporation in the biological processes. In addition, soils were rich in manganese (Mn) and iron (Fe) (Table 2). These essential micronutrients might thereby contribute to both enhanced enzymatic activities and an important nutrient supply playing a crucial role in the indri physiology [35]. Further, heavy metals found in the soil, such as cobalt (Co), chromium (Cr), copper (Cu), nickel (Ni) and zinc (Zn), were suitably below the threshold value for either ecological and health risks (Table 2) [36].

**Table 2:** Geophagic soil characteristics, average and standard error (es)

Geophagic soil		Average ± es
Granulometry	Clay	14.7 ± 1.4
	Silt	8.9 ± 0.9
	Sand	76.4 ± 1.2
	pH (H2O)	4.2 ± 0.1
Total carbon and nitrogen	N (%)	0.23 ± 0.02
	C (%)	3.18 ± 0.31
	C/N	13.71 ± 0.41
Pseudo total elements (mg/kg)	Al	77273 ± 5638
	Ca	427 ± 91
	Co	6.08 ± 1.61
	Cr	46.67 ± 9.64
	Cu	15.49 ± 4.48
	Fe	39394 ± 6102
	K	476 ± 192
	Mg	263 ± 108
	Mn	201 ± 61
	Mo	2.57 ± 0.58
	Na	104 ± 14
	Ni	17.35 ± 5.81
	P	287 ± 51
	Pb	39.93 ± 5.28
	S	279 ± 16
	Si	279 ± 98
	Sn	3.17 ± 0.28
	Ti	2212 ± 491
	V	67.42 ± 24.54
	Zn	55.51 ± 10.10
Dithionite-extractable metals (mg/kg)	Al	3580 ± 463
	Fe	7988 ± 852
	Ti	156 ± 37

### Mycobiome of the Geophagic Soil and Indri

**Fig. 2:**
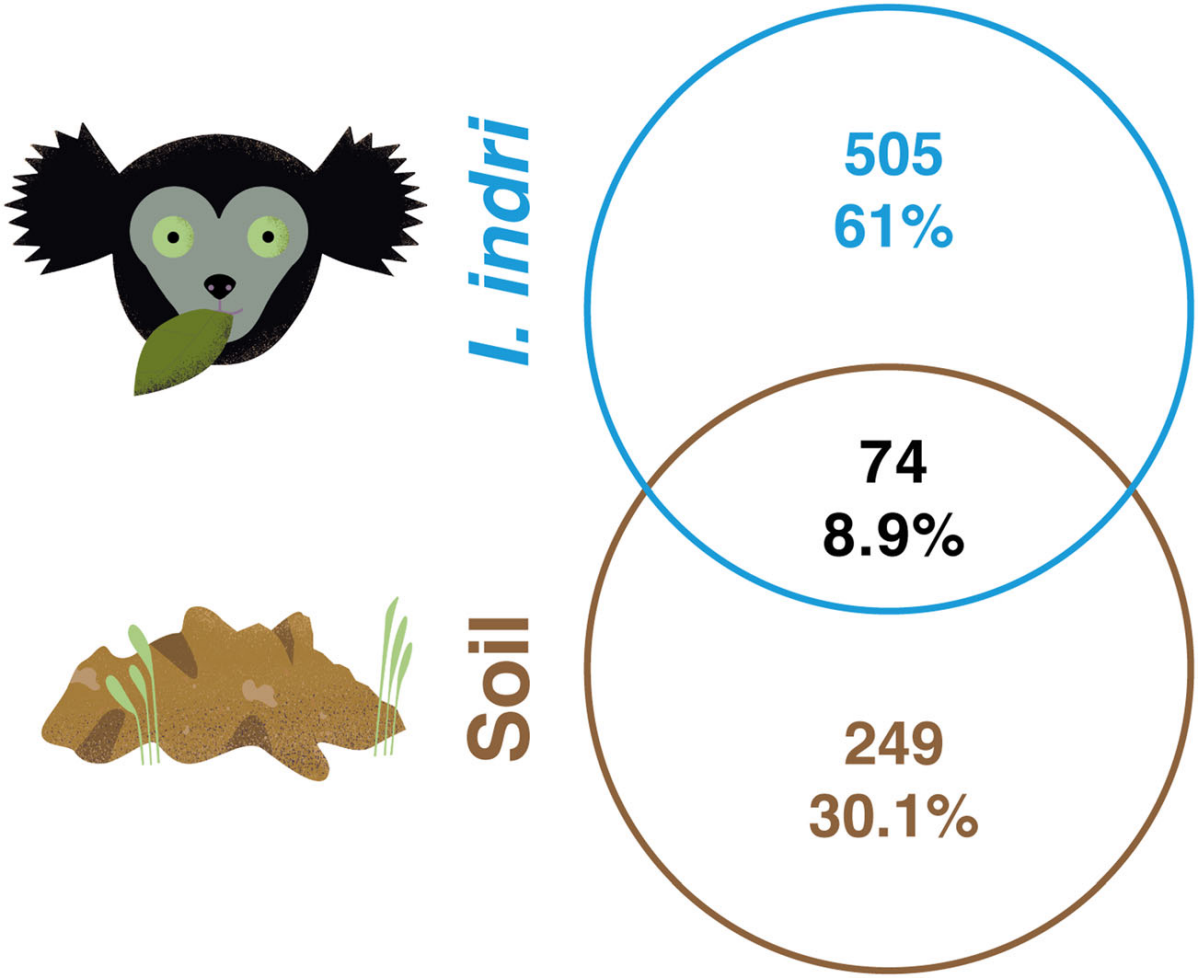
Venn diagram showing the number and percentage of shared fungal OTUs between geophagic soils and indri. OTUs were defined by 97% sequence similarity

After bioinformatics analysis, we obtained 437,872 reads clustered in 1110 OTUs (97% identity). Rarefaction curves showed that almost all the soil and indris faecal samples nearly reached plateau (Fig. S1). We found that 74 (8.9%) of the OTUs were shared between soil and indris’ faeces samples (Fig. 2). To the best of our knowledge, only another work has investigated the possible overlap between microbial species in the gut and soil [37]. The authors analysed more than 3000 samples, finding a low number of microbial classes shared between soil and gut. In addition, we re-analysed the OTU table of Tasnim et al. [37], and we found a considerably lower percentage (~ 2%) of shared OTUs (i.e. soil and gut) than in our dataset.

**Fig. 3:**
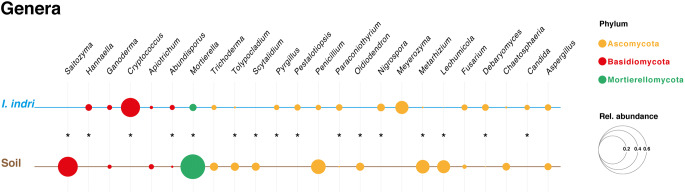
Bubble plot representing the relative abundance of the most abundant Genera. The asterisk (*) indicates the significative difference between soil and indri samples (*p* value < 0.05 and LDA score > 2.0)

Although with differences in relative abundance, some genera were found both in soil and indris’ faeces, including *Fusarium*, *Aspergillus*, *Penicillium*, *Apiotrichum*, *Ganoderma*, *Mortierella*, *Metarhizium*, *Tolypocladium* and *Chaetosphaeria* (Fig. 3). Several members affiliated to the genera *Fusarium, Aspergillus* and *Penicillium* have been commonly found in primates, especially with a vegetarian diet, as well as in forest soil and leaves of herbaceous and woody plants [10, 38, 39]*.* In some species of *Aspergillus* and *Penicillium* is reported the presence of catalytic enzymes such as pectin methyl esterase and polygalacturonase involved in plant polysaccharide degradation [39–41]. Besides, xylanase genes linked with the degradation of xylan, xylose and/or carboxymethyl cellulose have been detected in some *Fusarium* species [39, 42]. *Apiotrichum Mortierella* and *Ganoderma* are soil-associated genera involved in the decomposing of plant material, and some members may be associated with mammals [43–48]. Further, *Chaetosphaeria* is a cosmopolitan genus mainly found in the soil, rhizosphere or plant material [43, 49], and Metarhizium and Tolypocladium are entomopathogenic fungal taxa associated with soil-borne insects [50, 51].

On the contrary, *Candida* and *Cryptococcus* that are frequently detected in human and non-human primates’ gastrointestinal tracts were only present in faecal samples [10, 44, 52] (Fig. 3). A few species affiliated to *Cryptococcus* (i.e. *C. neoformans*) can cause Cryptococcosis, an animal-associated infectious disease with a worldwide distribution [53]. Further, these species can grow and proliferate in the decomposing wood of tree holes and the soils covered by plant debris [53–55]. Consequently, the pathogen can be spread among individuals via an environmental or zoophilic way [53, 55]. Although we are aware of the technical limitation (i.e. short reads), the OTU 2157 (with the highest frequency among *Cryptococcu*s OTUs) resulted in being the closest relative with *C. neoformans* (Fig. S2). The detection of this taxon could be seen as a health problem for potential overlap with humans, specifically for the rural communities present in the area.

Furthermore, fungal species only present in indris’ faeces were *Nigrospora* and *Meyerozyma*, which have been frequently found in association with primates, leaves and soil [47, 56] (Fig. 3).

**Fig. 4:**
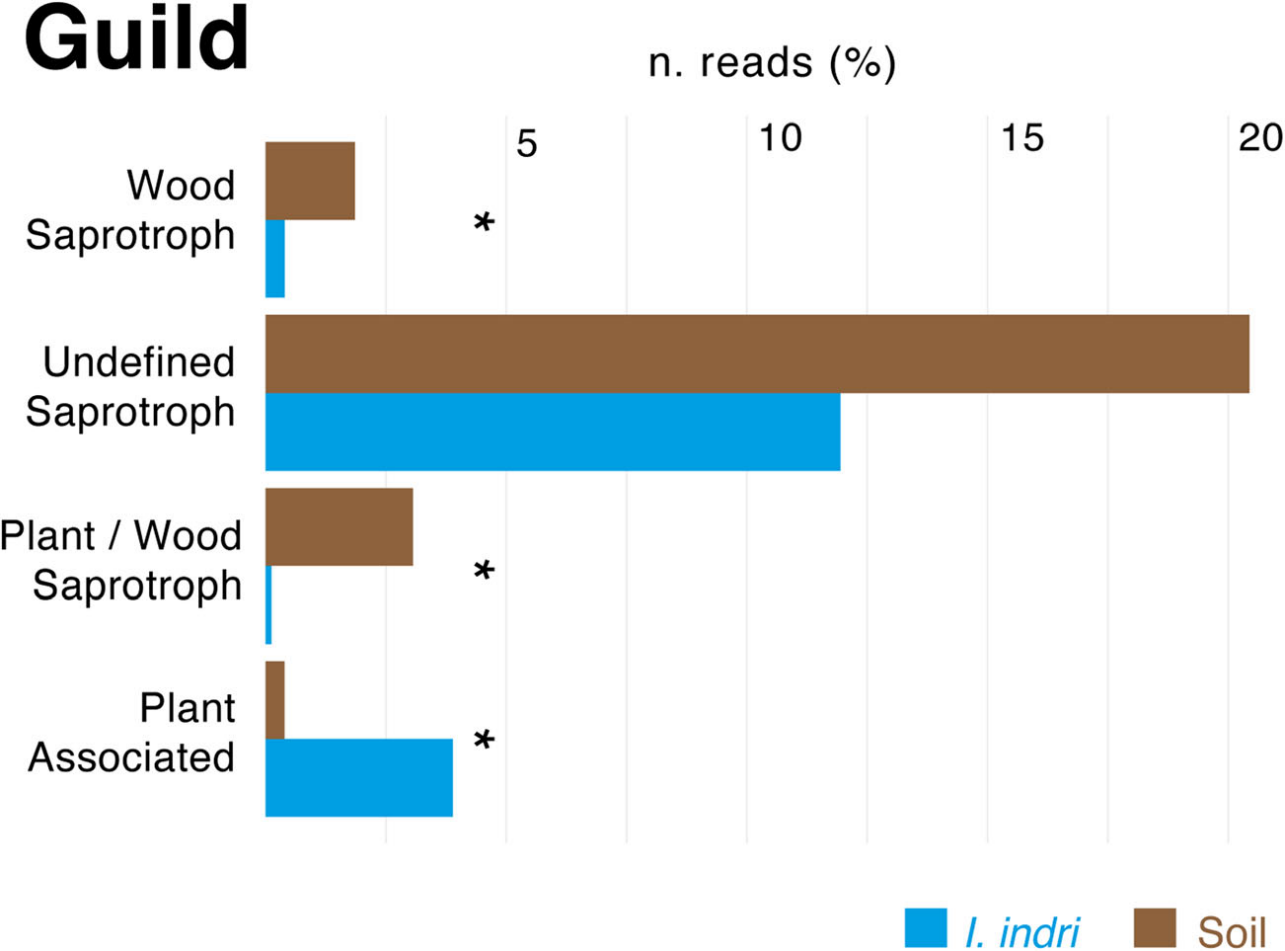
Bar plots representing the relative abundance of predicted fungal functions/guilds. The asterisks (*), indicate the significative difference between soil and indri samples (*p* value < 0.05 and LDA score > 2.0) LDA score and *p* value of the most abundant genera are shown in Supplemental information (Tab. S1 and S2)

The high percentage of ‘plant-associated’ fungi in the indris’ mycobiome is not surprising considering the linkages between the folivorous diet and the consequent accumulation of leaf-associated microbes in their gut (Fig. 4). For instance, these environmental fungi may survive, influence and, in some cases, colonise the gut [52]. Yet, the high percentage of ‘undefined saprotrophs’ fungal species may assist the breakdown of indigestible leaf cellulose and the redistribution of the nutrients [39, 57] (Fig. 4). In soil, saprophytic fungi are well known for the production of several secondary metabolites that play a crucial role in the initial destruction of complex organic compounds [58, 59]. Nevertheless, saprotrophic fungi could have a beneficial role in the production of enzymes necessary for the neutralisation of toxic compounds derived from the diet [60]. Although some environmental fungal species can be passengers or transient inhabitants of the indris’ gut, they most likely affect the gut microbiome directly or indirectly (i.e. interaction with other microbes) [8, 61]. During geophagy, indris assumes soil microorganisms, which probably can colonise the intestine, at least in part and transiently. Thus, they effectively could fulfil a specific temporary or stable physiological role (e.g. plant polysaccharides, detoxification and production of bioactive or antimicrobial compounds) [39, 57]. Therefore, we cannot exclude that the continuous intake of soil microorganisms through geophagy could constitute for indris a sort of ‘treatment’ that they seek, relevant for their health.

## Conclusion

Non-human primates are of particular interest for deepening our knowledge about bacterial microbiome research, but mycobiota of wild populations have been poorly explored. Recent findings have demonstrated the link between diet, habitat integrity and bacterial and fungal diversity in the host gut, rethinking the role of gut microbiota research as a tool for conservation [12, 62, 63]. As the microbial diversity may directly impact host health [64], the fungal diversity and the characteristics of the geophagic soil could play a crucial role in the indri’s health. Thus, the soil may be considered a source of some fungal species and essential nutrients [8]. With this regard, protecting the lemur habitat integrity may be reflected in protecting the integrity of gut microbial diversity, especially in specialist primates, like the leaf-eating indris. Our findings expand the current knowledge of the gut fungal diversity and geophagy in wild non-human primates that could be a baseline for further studies regarding the lemurs, including indris, conservation.

## Supplementary Information

ESM 1 (DOCX 2971 kb)

ESM 2 (BIOM 310 kb)
