# Supplementary material for: I Like the Way You Eat It: Lemur (Indri indri) Gut Mycobiome and Geophagy
Source: Microb Ecol. 2021 Jan 20;82(1):215–23. doi: 10.1007/s00248-020-01677-5 (PMC8282574; doi:10.1007/s00248-020-01677-5)
Supplement: Supplementary file 2 — (DOCX 2971 kb) [file 248_2020_1677_MOESM1_ESM.docx]

**Supplemental tables and figure**

**I like the way you eat it: Lemur (*Indri indri*) gut mycobiome and geophagy**

Luigimaria Borruso ^1^, Alice Checcucci ^2^, Valeria Torti ^3^, Federico Correa ^2^, Camillo Sandri ^2,4^, Daine Luise ^2^, Luciano Cavani ^2^, Monica Modesto ^2^, Caterina Spiezio ^4^, Tanja Mimmo ^1^, Stefano Cesco ^1^, Maura Di Vito ^5^, Francesca Bugli ^5,6^, Rose M. Randrianarison ^7,8^, Marco Gamba ^3^, Nianja J. Rarojoson ^9^, Cesare Avesani Zaborra ^4^, Paola Mattarelli ^2^, Paolo Trevisi ^2^, Cristina Giacoma ^3^

^1^Faculty of Science and Technology, Free University of Bolzano-Bozen, Piazza Università 5, 39100 Bolzano-Bozen, Italy

^2^Department of Agricultural and Food Sciences, University of Bologna, Viale Fanin 44, 40127 Bologna, Italy

^3^Department of Life Sciences and Systems Biology, University of Torino, Torino, Italy.

^4^Department of Animal Health Care and Management, Parco Natura Viva - Garda Zoological Park, Bussolengo, Verona, Italy

^5^Dipartimento di Scienze Biotecnologiche di Base, Cliniche Intensivologiche e Perioperatorie, Università Cattolica del Sacro Cuore, Largo A. Gemelli 8, 00168 Rome, Italy;

^6^Dipartimento di Scienze di Laboratorio e Infettivologiche, Fondazione Policlinico Universitario A. Gemelli IRCCS, Largo A. Gemelli 8, 00168 Rome, Italy

^7^Groupe d’Étude et de Recherche sur les Primates de Madagascar (GERP), Cité des Professeurs – Fort Duchesne, BP 779 – Antananarivo 101 – Madagascar

^8^Mention d'Anthropobiologie et de Développement Durable (MADD), Université de Antananarivo, Madagascar

^9^Laboratoire de Pédologie, FOFIFA à Tsimbazaza, BP.1690 Antananarivo.

Authors for correspondence

Luigimaria Borruso

[luigimaria.borruso@unibz.it](mailto:luigimaria.borruso@unibz.it)

Paola Mattarelli

paola.mattarelli@unibo.it

**Contents**

Figures of the rarefaction curves of the indri and soil samples (Figure S1) 2

Phylogenetic tree of the *Cryptococcus* group (Figure S2) 2

Table of the LefSe analysis identification of the most abundant fungal Genera (Table S1) 3

LefSe analysis identification of the fungal guild (Table S2) 3

**
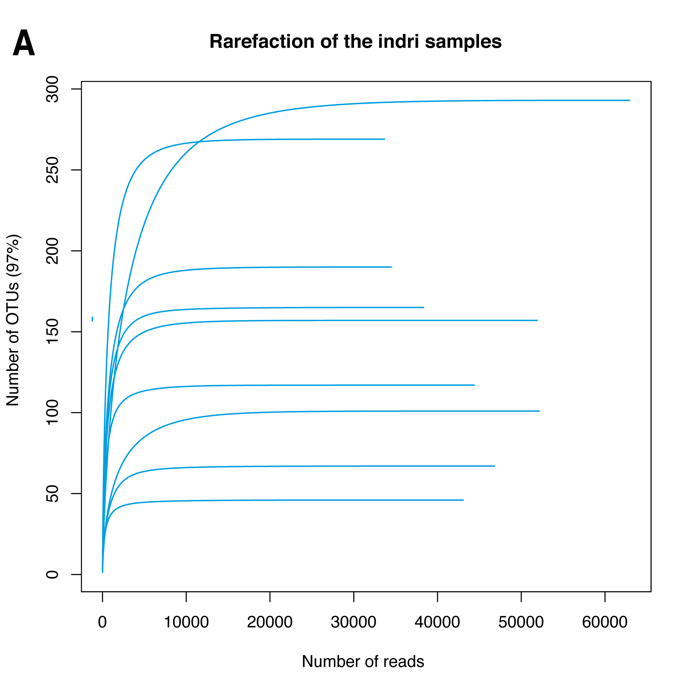
Fig. S1**: Rarefaction curves of the indris A) and soil B) samples

**
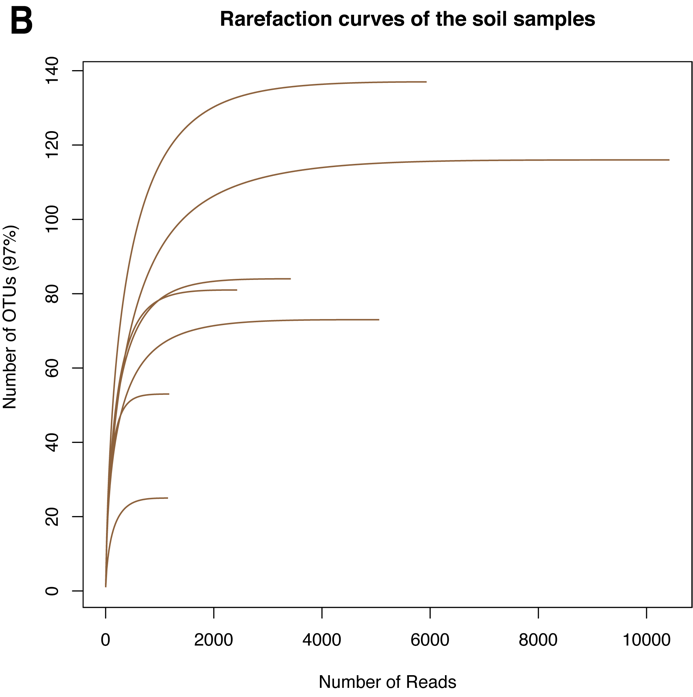
**

**Fig. S2**: Neighbour-joining phylogenetic tree of the *Cryptococcus* group and related OTU found in the indris’ samples. Bootstrap percentages from 1000 replications are shown on the branches. Type strain (T) and GenBank accession numbers are indicated after the species name. *C. rajasthanensis*, *C. aureus*, *C. flavescens* and *C. taibaiensis* are newly described as *Papiliotrema rajasthanensis*, *P. aurea*, *P. flavescens* and *Vishniacozyma taibaiensis* respectively [1].

**
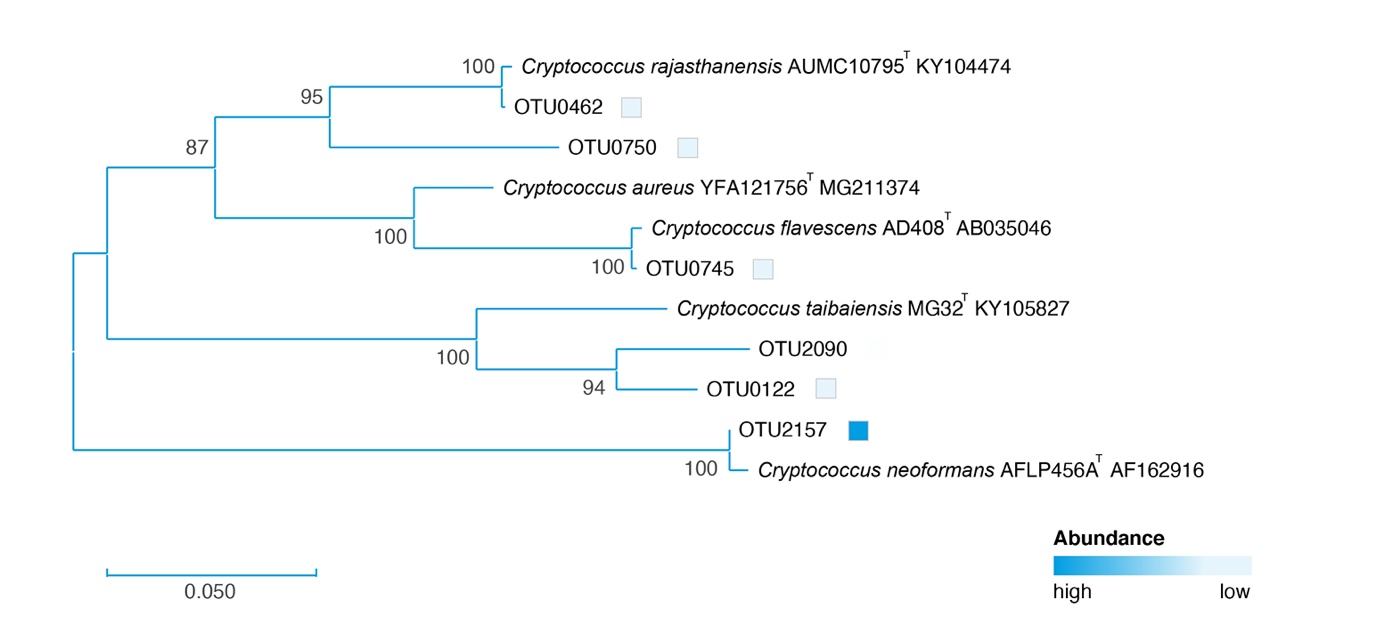
**

**Tab. S1**: LEfSe analysis identification of the most abundant fungal Genera.

| Genus |  | LDA score | *p* value |
| --- | --- | --- | --- |
| *Mortierella* | Soil | 3.8 | > 0.01 |
| *Saitozyma* | Soil | 3.7 | > 0.001 |
| *Cryptococcus* | Indri | 3.7 | > 0.01 |
| *Leohumicola* | Soil | 3.4 | > 0.001 |
| *Metarhizium* | Soil | 3.3 | > 0.001 |
| *Scytalidium* | Soil | 3.2 | > 0.001 |
| *Oidiodendron* | Soil | 3.2 | > 0.01 |
| *Tolypocladium* | Soil | 3.1 | > 0.01 |
| *Candida* | Indri | 3.0 | > 0.01 |
| *Nigrospora* | Indri | 3.0 | > 0.01 |
| *Pestalotiopsis* | Indri | 3.0 | > 0.01 |
| *Paraconiothyrium* | Indri | 2.9 | > 0.01 |
| *Abundisporus* | Indri | 2.9 | > 0.05 |
| *Hannaella* | Indri | 2.9 | > 0.05 |
| *Debaryomyces* | Indri | 2.9 | > 0.01 |
| *Pyrgillus* | Indri | 2.9 | > 0.01 |

**Tab. S2**: LEfSe analysis identification of the fungal guild. (Abbreviations: n.s., not significant).

| Functional guild | Class | LDA score | *p* value |
| --- | --- | --- | --- |
| Plant associated | Indri | 5.3 | 0.003 |
| Undefined Saprotroph | / | / | n.s. |
| Plant Saprotroph-Wood Saprotroph | Soil | 5.3 | 0.001 |
| Wood Saprotroph | Soil | 5.1 | 0.005 |

**Reference**

1. Liu XZ, Wang QM, Göker M, Groenewald M, Kachalkin A V., Lumbsch HT, et al. Towards an integrated phylogenetic classification of the Tremellomycetes. *Stud Mycol* 2015; **81**: 85–147.
